# Dipterocarpol in Oleoresin of *Dipterocarpus alatus* Attributed to Cytotoxicity and Apoptosis-Inducing Effect

**DOI:** 10.3390/molecules27103187

**Published:** 2022-05-17

**Authors:** Ploenthip Puthongking, Chawalit Yongram, Somporn Katekaew, Bunleu Sungthong, Natthida Weerapreeyakul

**Affiliations:** 1 Division of Pharmaceutical Chemistry, Faculty of Pharmaceutical Sciences, Khon Kaen University, Khon Kaen 40002, Thailand; pploenthip@kku.ac.th; 2 Division of Cannabis Health Science, College of Allied Health Sciences, Suansunandha Rajabhat University, Samut Songkhram 75000, Thailand; chawalit.yo@ssru.ac.th; 3 Department of Biochemistry, Faculty of Science, Khon Kaen University, Khon Kaen 40002, Thailand; somkat@kku.ac.th; 4 Integrative Pharmaceuticals and Innovation of Pharmaceutical Technology Research Unit, Faculty of Pharmacy, Mahasarakham University, Maha Sarakham 44150, Thailand; bunleu.s@msu.ac.th; 5 The Research Institute for Human High Performance and Health Promotion, Khon Kaen University, Khon Kaen 40002, Thailand

**Keywords:** dipterocarpol, oleo-resin, *Dipterocarpus alatus* Roxb. ex G. Don, apoptosis

## Abstract

*Dipterocarpus alatus* Roxb. ex G. Don is widely found in Southeast Asia. Its oleo-resin has reportedly been used in biodiesel production. Two different biodiesel production processes produce resinous byproducts, namely degumming (DG) and distillation (DT). Gas chromatography-mass spectrometry identified sesquiterpenes and triterpenes in oleo-resin, DG, and DT; and long-chain hydrocarbons in oleo-resin. High-performance liquid chromatography detected dipterocarpol as a marker compound, with the highest to lowest amounts detected in DG, DT, and oleo-resin, respectively. Oleo-resin, DG, and DT exerted more cytotoxicity than dipterocarpol, and melphalan, a chemotherapeutic drug. Oleo-resin, DG, and DT exerted cytotoxicity to a different degree in T cell leukemia (Jurkat), cervical adenocarcinoma (HeLa), and human hepatocellular carcinoma (HepG2) cells, while the highest selectivity was found in the Jurkat cells compared to the non-cancer Vero cells. Dipterocarpol exhibited the highest cytotoxicity in HepG2 cells and the lowest cytotoxicity in Jurkat cells. Oleo-resin, DG, and DT induced apoptosis in Jurkat cells. In oleo-resin, DG, and DT, dipterocarpol and other compounds may act in synergy leading to cytotoxicity and an apoptosis-inducing effect. Oleo-resin, DG, and DT could be potential sources for anticancer agents. Dipterocarpol could serve as a biomarker for follow ups on the anticancer activity of a sample from *D. alatus.*

## 1. Introduction

Plant resin may be generally referred to as sap or exudate and contains numerous substances, most commonly secondary metabolites or phenolic and terpenoid compounds. Plant resin is synthesized in epithelial cells and stored in secretory structures, namely resin canals or resin ducts found in roots, stems, leaves, flowers, and fruits [1]. There are two types of resin in the genus *Dipterocarpus*. The first type is liquid resin (oleo-resins), which contains resinous material and essential oils, and the second type is hard resin (dammar) that consists of sesquiterpenes and triterpenes [1,2].

*Dipterocarpus alatus* Roxb. ex G. Don (DA) is a non-food plant in the genus *Dipterocarpus*. Traditionally, in Southeast Asia, oleo-resin from DA was used as fuel in torches with rotten wood. In Burma, DA oleo-resin was used as waterproof enamel cloth to make umbrellas and to prepare Garjan or Kanyin oil [2]. In Thailand and Laos, it was used for caulking baskets and boats [3,4]. Industry uses of oleo-resin include the creation of varnish [5], preparation of lithographic inks [2], and as a fixative in perfumes [4]. The ethnomedicinal use of the DA oleo-resin, according to the Indian Ayurveda, was for the treatment of genitourinary diseases [6], externally for wound healing, and for counteracting putrefaction [5]. In Thailand, the oleo-resin of DA was investigated in the production of biodiesel with no harm to DA trees [7,8]. The oleo-resin was treated with a degumming reaction to separate oil and gum. The DA oleo-resin possessed an acceptable heating value, viscosity, density, flash point, pour point, and the corrosion of copper qualified as diesel fuel [7]. Additionally, DA oleo-resin was reported to produce gasoline-like-fuel (GLF) and diesel-like-fuel (DLF) following a fast pyrolysis process, and both GLF and DLF showed properties conforming to gasoline and diesel-fuel properties [8].

Cancer is one of the leading causes of death and the World Health Organization (WHO) expects an increase in the mortality rate in the world of nearly 80% by 2030 [9]. Additionally, DA oleo-resin exhibited decent biological activity. Dipterocarpol is a major compound that was previously reported in DA oleo-resin (67%) [10]. Dipterocarpol was reported to possess anti-human acyl-CoA: cholesterol acyltransferase (ACAT) against hACAT1 and hACAT2 with IC_50_ values of 12.4 and 30.5 µM, respectively [11]. Dipterocarpol exerted strong cytotoxicity on epidermoid cancer KB cells [12] and antibacterial activity against *Streptococcus pyogenes* [13]. Moreover, dammarenolic acid, which was found at a proportion of 23% in dammar resin—resin obtained from the family Dipterocarpaceae—showed the highest activity against HL-60TB (leukemia), HOP-62 (lung cancer), and COLO 205 (colon cancer) [14]. Despite, many previous studies on the cytotoxicity of the phytoconstituents of the genus *Dipterocarpus*, the effect of the DA oleo-resin on cancer cells has not yet been elucidated. The objective of this study was, therefore, to evaluate the antiproliferative activity and the mechanism of anticancer action of DA oleo-resin and its resinous by-products. Moreover, the marker compound dipterocarpol was analyzed with High Performance Liquid Chromatography (HPLC) to determine its content and contribution to the biological activity studied.

## 2. Results

### 2.1. Phytochemical Screening

Alkaloids, steroids, tannins, xanthones, saponins, and reducing sugar, which are secondary metabolites, were evaluated in oleo-resin, resin from degumming, and the distillation processes for *D. alatus* (Table 1). The results illustrated that the steroids group was only positively presented in oleo-resin, DG, and DT. Our result was in agreement with a previous study regarding the presence of sesquiterpenes and triterpenes in the oleo-resin from the genus *Dipterocarpus* [15].

### 2.2. GC-MS Analysis

Based on the phytochemical analysis result, the steroid group of the compound was found in all three samples. To expand on the description of the phytochemical results, the chemical structures were further elucidated by using GC-MS. Table 2 presents the chemical composition of oleo-resin, DG, and DT. The oleo-resin comprised of sesquiterpenes (63.23%), triterpenes (17.05%), and long-chain hydrocarbons C_15_–C_23_ (10.60%). Sesquiterpenes were mainly composed of α-gurjunene (30.31%) and (-)-isoledene (13.69%), which has previously been reported [16]. Resin from degumming (DG) contained sesquiterpenes (6.90%) and triterpenes (12.05%) (Table 2). Sesquiterpenes in DG mainly comprised calarene (4.43%), valerenal (1.06%), and unknown triterpenes C_30_ (Supplementary Table S1). Resin from distillation (DT) contained sesquiterpenes (2.04%) and triterpenes (16.93%) (Table 2). The triterpenes identified were mainly unknown triterpenes C_30_ (Supplementary Table S1). However, both DG and DT showed a higher content of triterpenes than sesquiterpenes compared to oleo-resin. Sesquiterpenes might be removed from the oleo-resin during the biodiesel production processes. The chemical compositions of oleo-resin, DG and DT are shown in detail in the Supplementary Materials (Supplementary Table S1).

**Figure 1:**
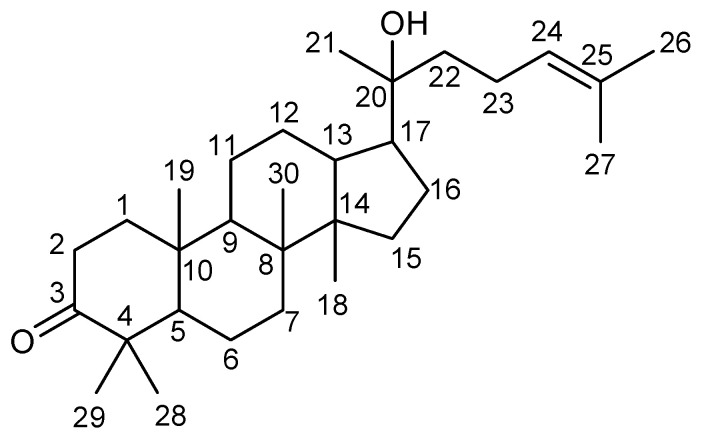
Structure of dipterocarpol isolated from oleo-resin.

Dipterocarpol (Figure 1) was isolated from oleo-resin in the form of white crystal. It was previously isolated from oleo-resin of *D. alatus* [14]. The ^1^H-NMR spectrum of dipterocarpol showed eight methyl signals at δ_H_ 1.69, (CH_3_-27), 1.63, (CH_3_-26), 1.15, (CH_3_-21), 1.08, (CH_3_-29), 1.04, (CH_3_-28), 1.00, (CH_3_-30), 0.94, (CH_3_-19), and 0.89, (CH_3_-18) ppm. Ten methylene proton signals of CH_2_-1, -2, -6, -7, -11, -12, -15, -16, -22, and -23, five methine proton signals of CH-5, -9, -13 and -17, and twenty-four characteristic peaks of the dipterocarpol structure were identified. Additionally, quaternary carbonyl carbon of C-3 was observed at δ_C_ 217.90 ppm, while the quaternary carbon of C-20 and C-25 was observed at δ_C_ 75.35 and 131.63 ppm, respectively. The ^1^H- and ^13^C-NMR spectra showed characteristic signals of dipterocarpol (Supplementary Table S2) similar to those reported in the previous study of Smirnova et al. (2012) [14]. Additionally, the isolated dipterocarpol was further used as the standard in this experiment.

### 2.3. Dipterocarpol Contents

Our results revealed dipterocarpol contents in oleo-resin, DG, and DT at 53.9 ± 2.5, 260.4 ± 2.9, and 162.7 ± 1.9 mg/g dry residue, respectively (Table 3). The dipterocarpol content was calculated from the standard curve of dipterocarpol in the range of 10–1000 µg/mL (y = 1.6566x − 0.0131; R² = 0.9994). The values of the limit of detection (LOD) and the limit of quantification (LOQ) of dipterocarpol, with reverse-phase HPLC, were 0.28 µg/mL, and 0.84 µg/mL, respectively. The precision of the proposed method expressed as % relative standard deviation (%RSD) was less than 1.23% for intra-day and 1.77% for inter-day (at concentrations of 100 and 700 µg/mL). The %recovery when using the spiked-sample method was 87.58%, which indicated high-efficiency extraction during sample preparation.

### 2.4. Cytotoxicity

The cytotoxicity of oleo-resin and resin from different preparation processes were studied in four cell lines of three cancer-cell types (liver, cervix, and leukemia) and noncancerous Vero cells. Melphalan, a chemotherapeutic drug, was used as a positive control. The results in Table 4 reveal the significantly high cytotoxicity of oleo-resin, DG, and DT against Jurkat, HeLa, and HepG2 cells, respectively. Interestingly, oleo-resin, DG, and DT exerted higher cytotoxicity in HeLa and Jurkat cells than in HepG2 cancer cells. The cytotoxicity of oleo-resin, DG, and DT in HepG2 and Jurkat cells was lower than in melphalan. The selectivity index was determined to indicate the selective cytotoxic effect in cancer cells and safety of the noncancerous Vero cells [17]. Oleo-resin, DG, and DT showed a greater selective index in Jurkat than HepG2 and Hela cells.

The isolated dipterocarpol from oleo-resin exhibited a greater cytotoxic effect in HepG2 and HeLa cell lines with respective IC_50_ values of 24.2 ± 0.9 µg/mL and 41.1 ± 4.0 µg/mL, but was inactive even when using a concentration of up to 221.4 µg/mL in the Jurkat cell line. Hence, dipterocarpol was dominantly attributed to the antiproliferative effect of oleoresin in HepG2 and HeLa cell lines but not in the Jurkat cell line. Moreover, dipterocarpol possesses a higher selective cytotoxicity against HepG2 (SI = 3.5) than melphalan (SI = 0.8), while melphalan possesses higher selective cytotoxicity against Jurkat (SI = 4.4) than dipterocarpol (SI = 0.4). The results suggested that the other existing compounds in oleo-resin, DG, and DT may be responsible for cytotoxicity in addition to dipterocarpol.

### 2.5. Determination the Mode of Cell Death

**Figure 2:**
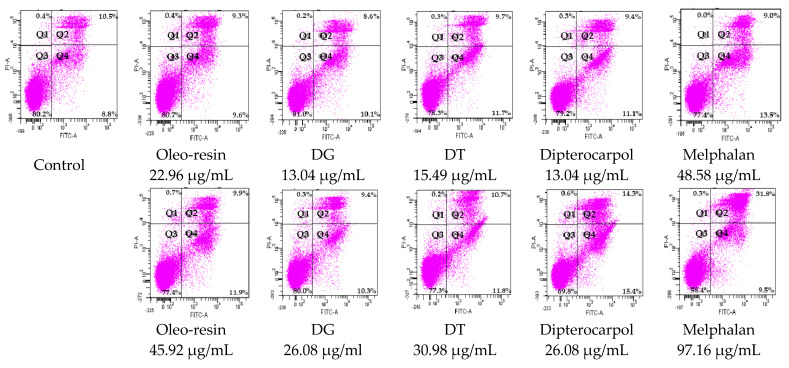
Cell dot plot represented the mode of cell death of oleo-resin and resin from different preparation processes and dipterocarpol at 1 × IC_50_ and 2 × IC_50_ concentrations after treatment for 24 h. Necrosis, late apoptosis, viable cell, and early apoptosis were shown in quadrant 1 (Q1) to 4 (Q4), respectively.

Oleo-resin, DG, and DT exerted high selectivity (SI > 3) in the Jurkat cell line over the Vero non-cancer cell line. Furthermore, we decided to study whether these test compounds induced cell death via apoptosis, which is a desirable action for a chemotherapeutic agent. The mode of cell death of oleo-resin, DG, DT, and dipterocarpol against the Jurkat cancer cell line was determined by using the Annexin V and propidium iodide (PI) kit after cells were treated for 24 h. If cells died via necrosis at less than 1%, the induction of apoptosis would be apparent [18]. According to the results in Table 5 and Figure 2, the rank order of the test compounds from a high to low percentage of total apoptosis (early and late stage of apoptosis) in the Jurkat cells was as follows: melphalan, dipterocarpol, DT, oleo-resin, and DG, respectively. The oleo-resin, DG, and DT showed a %total apoptosis from 18.8 to 22.5% with necrosis of less than 1%. Dipterocarpol induced the highest %apoptotic cell death compared to other test compounds, and this effect increased at a higher concentration. Our study presented that oleo-resin, DG, DT, and dipterocarpol act as cytotoxic agents, which induced Jurkat cells to undergo apoptosis cell death.

## 3. Discussion

In this study, we showed that dipterocarpol was found in oleo-resin and its byproducts, with the highest content found in resin from degumming (DG) followed by resin from distillation (DT), and oleo-resin. The crude plant oleo-resin may consist of common impurities such as phospholipids, sugars, acylglycerols, free fatty acids, steroids, and trace metals. The degumming process is a process that removes phospholipids and waxes among other products that are contained in oleo-resin [19]. Additionally, the distillation processes separate impurities from oleo-resin. It is possible that both biodiesel preparation processes can extract the dipterocarpol from oleo-resin. Hence, a lower dipterocarpol content was detected in oleo-resin compared to DG and DT (Table 2).

The GC-MS analysis identified sesquiterpenes and triterpenes in oleo-resin, DG, and DT. The resin of plants in Dipterocarpaceae has previously been reported to be composed of sesquiterpenes [20]. Moreover, archaeological resins of the family Dipterocarpaceae showed the presence of triterpenoid compounds [21], thereby indicating the stability of triterpenes. In our study, DG and DT were prepared by heating the oleo-resin to 100 °C, and 350 °C, respectively. Sesquiterpenes were found in a lower amount in both DG and DT compared to triterpenes. Moreover, the GC-MS analysis identified long-chain hydrocarbons in oleo-resin, which is in agreement with previous reports. The hexane fraction of bark from *Vateria copallifera* (Dipterocarpaceae) contained alkane hydrocarbon derivatives such as undecane, decane and dodecane [22]. The resin of *Shorea robusta* contained non-polar hydro-carbons and polycyclic aromatic hydrocarbon naphthalene [23].

Dipterocarpaceae resins have been reported to possess several biological activities such as wound healing [24] and analgesic activity [25]. Asiatic acid isolated from *Shorea robusta* resin possessed spermicidal and microbicidal activity [26]. Seven out of twenty compounds isolated from the dammar resin of *S. javanica* showed potent cytotoxic activity against HL60, and CRL1579 cancer cell lines and eleven compounds potentially inhibited tumor promotion on EBV-Ea activation induced by TPA [27].

In our study, the cytotoxic activity and selectivity of *D. alatus* could be classified into five categories [17]. First, the potentially cytotoxic compounds (IC_50_ < 100; SI ≥ 3) were oleo-resin, DG, DT, and melphalan against Jurkat; dipterocarpol against HepG2; and melphalan against HeLa cells. Second, moderate cytotoxic compounds (IC_50_ ≤ 1000; SI < 3) were oleo-resin, DG, DT, and melphalan against HepG2; oleo-resin, DG, DT, and dipterocarpol against HeLa cells. Third, the more toxic compound against normal Vero cells than for the Jurkat cell line was dipterocarpol. Previously, dipterocarpol was reported to be cytotoxic against RPMI-8226 (319.7 µM), K-562 (386.0 µM), and KB (14.37 µg/mL) cancer cell lines [12].

The chemical constituents in oleo-resin in the genus *Dipterocarpus* were sesquiterpenes and triterpenes. The reported sesquiterpenes were α-gurjunene, alloaromadendrene, β-gurjunene, caryophyllene, humulene [20,28], (-)-7β,10α-Selina-4,11-diene, and α-selinene [29]. The reported triterpenes were asiatic acid, ocotillone [12,30], and dipterocarpol [30]. Alloaromadendrene and α-gurjunene [20] showed no anticancer activity. Dipterocarpol, found in *D. alatus*, has previously been reported to have cytotoxic activity against human epidermoid cancer KB tumor cells, DU-145, RPMI-8226 and K-562 cancer cell lines [10,12,14]. Terpenes found in the genus *Dipterocarpus* have shown potential cytotoxicity against several cancer cell lines [31].

Our GC-MS analysis suggests that oleo-resin contains more than 1% of α-gurjunene, β-caryophyllene, calarene, (-)-isoledene, alloaromadendrene, γ-gurjunene, spathulenol, α-copaen-11-ol, valerenal, methyl hexadecanoate, methyl linoleate, methyl oleate, and unknown triterpene C30 compounds [16]. Cytotoxicity of β-caryophyllene was reported against A549, HT-29, UACC-62, and K562 [32]. Isoledene induced apoptosis in HCT 116 cells [33]. Methyl linoleate demonstrated moderate cytotoxicity against the K562 cell line [34], and methyl oleate showed moderate cytotoxicity against the MCF-7 and HT-29 cell lines [35]. Alloaromadendrene inhibited the neoplastic +SA mouse mammary epithelial cells [36], and spathulenol showed cytotoxicity against MCF-7, OVCAR-3, and HaCaT cells [37]. In addition, α-gurjunene showed antibacterial and antifungal activities [38]. According to our study, it seems that oleo-resin, DG, and DT may consist of many bioactive compounds including terpenes that inhibit cancer cell growth. Both resinous byproducts from biodiesel production (DG and DT) were found to comprise more triterpenes than sesquiterpenes. The cytotoxic activity of DG and DT in the HepG2, HeLa, and Jurkat cancer cell lines may be mostly attributed to triterpenes.

Apoptosis has been known to be an end point of anticancer mechanisms [39]. Cell death by apoptosis is induced by a specific stimulus and occurs without the release of inflammatory mediators [40]. Most clinically used anticancer drugs exploit the apoptotic signaling pathways to induce cancer cell death [39]. In our study, oleo-resin, DG, and DT induced greater apoptosis than necrosis in Jurkat cell lines. Although dipterocarpol showed no cytotoxicity against Jurkat cells, increased concentrations of dipterocarpol (2 × IC_50_) induced apoptosis in Jurkat cells higher than that of oleo-resin, DG, and DT. The ROS level might be the cause for the induction of the apoptosis of dipterocarpol. It has been reported that triterpenes increased the pro-apoptotic capacity of the MCF-7 cell via a reduced intracellular ROS level, prevented H_2_O_2_-induced oxidative injury, and strongly induced apoptosis in MDA-MB-231 and U937 cells [41]. A previous study showed that oleanolic acid, a triterpene from *D. zeylanicus*, increased caspase 3/CED3 activity and ROS levels, caused DNA fragmentation, and apoptotic body formation resulting in oxidative stress, and apoptosis cell death in the filarial parasite *S. digitata* in vitro [42].

## 4. Materials and Methods

### 4.1. Chemicals

All organic solvents, HCL and H_2_SO_4_ used for the extraction were obtained from RCI Labscan (Samut Sakhon, Thailand). Sodium Hydroxide and FeCl_3_ were purchased from Q RëC™ (New Zealand). KOH was purchased from KemAus™ (Cherrybrook, Australia). Dragendorff’s reagent and sodium-potassium tartrate were purchased from Merck (Darmstadt, Germany). DMSO was purchased from Lab-Scan. Dulbecco’s modified Eagle’s medium (DMEM), RPMI Media 1640, 0.25% trypsin-EDTA (1×), fetal bovine serum (FBS), penicillin, and streptomycin were purchased from GIBCO^®^ (Invitrogen, Grand Island, NY, USA). FITC-conjugated Annexin V and propidium iodide (PI) were purchased from BioLegend (San Diego, CA, USA). Neutral red (NR) was purchased from Sigma-Aldrich (Saint Louis, MO, USA). TLC silica gel 60 F_254_ and silica gel 60 (grain fraction 0.2–0.5 mm) were purchased from Merck (Darmstadt, Germany).

### 4.2. Materials

The oleo-resin of *Dipterocarpus alatus* Roxb. ex G. Don were collected from Phitsanulok province, Thailand during June 2014. The *D. alatus* (No. PSKKF03682) herbarium was identified according to botanical characters of *D. alatus* from Pooma, 1996 [3], by Associate Professor Suppachai Tiyaworanant, Faculty of Pharmaceutical Sciences, Khon Kaen University, Thailand. The resin from the degumming process was a byproduct from the oil production of oleo-resin. Briefly, oleo-resin (400 mL) was heated at 100 °C in 6% sodium hydroxide in water (100 mL) and stirred at 100 °C for 20 min. After that, it was cooled at room temperature for 24 h to separate oil and the mass of resinous byproduct from the degumming process (DG) (9.95% *w*/*w* yield). The second type of resinous byproduct was also prepared at 350 °C in the oil distiller under atmospheric pressure to obtain oil and the byproduct using the distillation process (DT) (30–40% *w*/*w* yield) [8]. 

### 4.3. Phytochemical Screening of Oleo-Resin and Resin from Different Preparation Processes

The phytochemicals screening of oleo-resin and resin from different preparation processes from *D. alatus* was adapted as per Srisongkram et al. (2022) [43]. Briefly, samples were prepared at concentration of 15 mg/mL in ethanol for tannins, xanthones, saponins and 15 mg/mL in chloroform for the steroids test and 30 mg/mL in ethanol for the alkaloids and reducing sugar tests. For tannin, the sample solution was incubated in a water bath for 10 min. Then, 15% FeCl_3_ was added in the supernatant. The dark green or blue–black color indicates the presence of tannin. For xanthones, the sample solution was incubated in a water bath for 10 min and centrifuged to then test the supernatant with 100 µL of 5% KOH. The yellow precipitate indicated the presence of xanthones. For saponin, the sample solution was shaken at room temperature for 1 h. After that, the persistence of frothing or bubbles was observed to detect the presence of saponin. For steroids, the sample solution was added with 1 to 2 drops of Conc. H_2_SO_4_. Steroids produced a red color in the lower chloroform layer. For alkaloids, the sample solution was mixed with 2 mL of 1% HCl and drops of Dragendorff’s reagent. A reddish brown precipitate with turbidity indicated the presence of alkaloids. For reducing sugar, 30 mg of crude dry residue was mixed with 1 mL of Fehling’s solution prepared from solution A and B at a ratio of 1:1. Solution A was a mixture of 7 g of copper II sulfate in 100 mL of water. Solution B was a mixture of 0.44 g sodium-potassium tartrate and 13.0 g sodium hydroxide in 100 mL of water. The reaction mixture was heated in a water bath for 10 min. The positive result of reducing sugar in glycosides was made apparent by the appearance of brick-red precipitate.

### 4.4. Phytochemical Identification

Then, 25.04 g of oleo-resin was purified using open column chromatography on silica and eluted with increasing polarity from 100% hexane to 100% ethyl acetate (EtOAc) and 100% methanol (MeOH) to produce 13 fractions (OA1-OA13) (Supplementary Figure S1). Fraction OA4 (5.67 g) was separated by column chromatography on silica and eluted with a solvent mixture of hexane and EtOAc at various ratios (0:100, 95:5, 90:10, 80:20, 70:30, 0:100 *v*/*v*), following with 100% MeOH to generate nine additional fractions (OB1-OB9). Fraction OB5 was purified by recrystallization with methanol to obtain pure compound 1 (1.01 g, 4.06% yield). The NMR spectra of each isolated compound was recorded in CDCl_3_ on a Varian Mercury Plus spectrometer operating at 400 mHz (^1^H) and 100 mHz (^13^C) (Varian, Inc., Palo Alto, CA, USA).

Compound 1 (dipterocarpol): as a white crystal, ^1^H-NMR (CDCl_3_, 400 MHz) δ ppm: 5.12, (1H, m, CH-24), 2.47, (2H, m, CH_2_-2), 1.96, (2H, m, CH_2_-23), 1.83, (2H, m, CH_2_-1), 1.78 and 1.20, (2H, m, CH_2_-16), 1.69, (3H, brs, CH_3_-27), 1.66, (1H, m, CH-17), 1.63, (3H, brs, CH_3_-26), 1.48 and 1.38, (2H, m, CH_2_-6), 1.47 and 1.22, (2H, m, CH_2_-7), 1.43, (6H, m, CH_2_-11, -12, -15), 1.39 (3H, m, CH-9 and CH_2_-22), 1.29 (1H, m, CH-5), 1.15, (3H, s, CH_3_-21), 1.08, (3H, s, CH_3_-29), 1.04, (3H, s, CH_3_-28), 1.00, (3H, s, CH_3_-30), 0.94, (3H, s, CH_3_-19), 0.89, (3H, s, CH_3_-18). ^13^C-NMR (CDCl_3_, 100 MHz) δ ppm: 217.90 (C-3), 131.63 (C-25), 124.68 (C-24), 75.35 (C-20), 55.32 (C-5), 50.25 (C-14), 49.78 (C-17), 47.42 (C-4), 42.36 (C-13), 40.46 (C-22 and C-8), 40.26 (C-7), 39.88 (C-1), 36.81 (C-9), 34.52 (C-6), 34.11 (C-2), 31.15 (C-15), 27.51 (C-12), 26.69 (C-29), 25.76 (C-27), 25.47 (C-21), 24.79 (C-16), 22.55 (C-23), 22.02 (C-11), 21.01 (C-28), 19.64 (C-10), 17.72 (C-26), 16.34 (C-18), 16.03 (C-19), 15.20 (C-30). IR (film; V_max_): 3544, 2940, 2865, 1701, 1454, 1389, 1376 cm^−1^.

### 4.5. Cell Culture

The human hepatocellular carcinoma (HepG2), cervical adenocarcinoma (HeLa) and normal African green monkey kidney (Vero) cells lines were cultured in Dulbecco’s Modified Eagle Medium (DMEM), supplemented with 10% fetal bovine serum (FBS), while human acute T cell leukemia (Jurkat) was cultured in an RPMI medium with 10% FBS. Then, 1% penicillin/streptomycin was added to the mediums and cells were cultured at 37 °C in 5% atmospheric CO_2_, and 95% humidity. The HepG2 (ATCC# HB-8065), and Vero (ATCC#CCL-81) were obtained from American Type Culture Collection (Manassas, VA, USA). The Jurkat, and Hela cell lines were obtained from Center for Research and Development of Medical Diagnostic Laboratories (CMDL), Faculty of Associated Medical Sciences, Khon Kaen University, Thailand.

### 4.6. Antiproliferative Activity

The antiproliferative effects of oleo-resin and resin from different preparation processes in cancer cell lines were determined using a neutral red assay as previously reported by Jantamat et al. (2019) [17] and Srisongkram et al. (2019) [44]. Cells were seeded in a 96-well plate (3 × 10^5^ cells/mL for Vero and HepG2; 4 × 10^5^ cells/mL for HeLa, and 5 × 10^5^ cells/mL for Jurkat). The dry residue was dissolved with DMSO and diluted with the media. The final concentration of DMSO was less than 1% *v*/*v*. The test compounds (100 μL) with varied concentrations, from 10–500 μg/mL, were added into each well and incubated for 24 h. Then, the cells were washed with PBS and 100 μL of 50 μg/mL of Neutral Red was added and incubated for 2 h. After washing with PBS, cells were lyzed with 0.33% HCl in isopropanol. The absorbance of NR was measured at 537 nm and 650 nm for the reference wavelength using a microplate reader. Cells with no treatment or untreated cells (control) were used as negative controls. Melphalan was used as the positive control. The IC_50_ was determined by plotting %cell cytotoxicity versus the concentrations of samples. The selectivity index (SI) value was calculated with the ratio of the IC_50_ value of a normal cell versus cancer cell.

### 4.7. Mode of Cell Death

The modes of cell death of oleo-resin, DG, DT and dipterocarpol against Jurkat cancer cell lines were determined using the Annexin V and propidium iodide (PI) kit. Cells (1 × 10^6^ cells/mL) were seeded in a 24-well plate and incubated for 24 h. Cells were treated with 1 × IC_50_ and 2 × IC_50_ of oleo-resin, DG, DT and dipterocarpol for 24 h. Cells were washed with cold BioLegend’s cell-staining buffer and resuspended in the binding buffer. Then, the cells were stained with Annexin V and PI and were incubated in the dark. After that, the cells were resuspended in the binding buffer and analyzed using the BD FACSCantoII Flow cytometer (BD Biosciences, San Jose, CA, USA). The sample data were analyzed using BD FACSDiva software Version 6.1.3 (BD Biosciences, San Jose, CA, USA) [45]. The untreated cells were the control. Melphalan was used as the positive control.

### 4.8. HPLC Analysis of Dipterocarpol

This assay was adapted from Yomgram et al. (2021) [46]. The RP-HPLC analysis of dipterocarpol was performed using HPLC auto injection (Primaide 1000 series, Hitachi, Japan) coupled with a photodiode array detector, column (00F-4760-E0 Luna^®^ 3 µm Polar C_18_ 100 Å, 150 × 4.6 mm) (Phenomenex, Torrance, CA, USA). The mobile phase consisted of acetonitrile (solvent A) and 0.05% trifluoroacetic acid (*v*/*v*) (solvent B) at a ratio of 9:1 (solvent A: solvent B) for isocratic, from 0 to 15 min at a flow rate 1 mL/min, the column temperature was 25 °C and the injection volume was 20 μL. The respective UV-diode array detection wavelength was 210 nm. The HPLC chromatograms of oleo-resin, DG, DT and dipterocarpol dissolved in methanol were compared. The presence of dipterocarpol in oleo-resin, DG and DT was detected based on the retention time. Analytical method validation was performed in accordance with the International Council for Harmonisation guideline, ICH guideline Q2(R1) [47]. The isolated dipterocarpol was characterized (in Section 4.4) and further used as a reference standard.

### 4.9. GC-MS Analysis

The chemical composition of oleo-resin and resin from different preparation processes was determine by GC-MS (SHIMADZU QP-2010) (Kyoto, Japan). The mass detector was operated in the electron ionization (EI) mode. The column flow was 1.00 mL/min. Carrier gas helium was 5.5; the ionization energy was 70 eV; and the split/splitless ratio was set to 1:30 after 45 s. The samples (1 µL) were injected into the Agilent J&W VF-5ms column (30 m × 0.25 mm, 0.25 µm) (Santa Clara, CA, USA). The temperature of the injector was maintained at 290 °C and held for 95 min. The oven temperature was initially set at 60 °C (hold time 3 min), with a gradient from 60 to 120 °C (3.0 °C/min, hold 1 min), 120 to 280 °C (2 °C/min, hold 1 min), and from 280 to 300 °C (10 °C/min, hold 2 min). The peak was analyzed based on the GC retention time and a mass spectral comparison was performed with the NIST147.LIB and WILEY7.LIB library [48].

### 4.10. Statistical Analysis

The results were compared by using a one-way ANOVA with SPSS 19.0 software (SPSS Inc., Chicago, IL, USA). Tukey’s honest significant-difference test was conducted to determine the significant differences between samples (*p* < 0.05).

## 5. Conclusions

In conclusion, oleo-resin, DG, and DT exhibited a different degree of cytotoxicity in HepG2, HeLa, and Jurkat cells. Dipterocarpol, which belongs to the triterpenes group, was found in DG at a higher amount than in DT and oleo-resin, respectively. Dipterocarpol exerted higher cytotoxicity in HepG2 cells, followed by HeLa, and Jurkat cells, while oleo-resin, DG, and DT had the highest cytotoxicity in Jurkat cells followed by HeLa, and HepG2 cells. The cytotoxic effect of DG was higher than that of DT and oleo-resin in Jurkat cells, and this rank order (DG > DT > oleo-resin) was similarly observed in HeLa and HepG2 cells. The results suggest that not only dipterocarpol, but also some other compounds including terpenes, were partially responsible for the anticancer effect of oleo-resin, DG, and DT. Oleo-resin, DG, and DT are promising sources of anticancer agents due to the presence of dipterocarpol as a marker for material standardization in the future.

## Figures and Tables

**Table 1:** Phytochemical screening of oleo-resin and resin from different preparation processes.

*D. alatus*	Chemical Groups					
	Alkaloids	Steroids	Tannins	Xanthones	Saponins	Reducing Sugar
Oleo-resin	-	+	-	-	-	-
	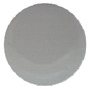	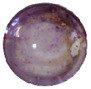	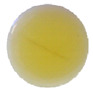	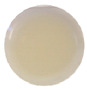	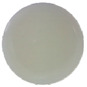	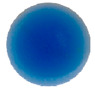
Byproduct from degumming process (DG)	-	+	-	-	-	-
	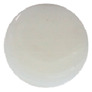	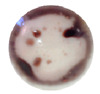	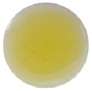	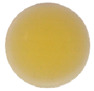	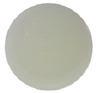	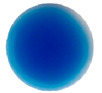
Byproduct from distillation process (DT)	-	+	-	-	-	-
	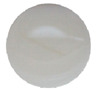	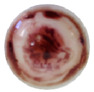	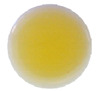	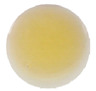	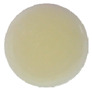	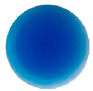
Positive results	Reddish brown precipitate	Red-color in lower chloroform layer	Dark green or blue-black color	Yellow precipitate	Frothing or bubbles	brick-red precipitate

**Table 2:** Chemical composition of oleo-resin and resin from different preparation processes during GC-MS analysis.

Chemical Composition	Relative Abundance (%)		
	Oleo-Resin	Byproduct from Degumming Process (DG)	Byproduct from Distillation Process (DT)
Sesquiterpenes (C_15_)	63.23	6.90	2.04
Triterpenes (C_30_)	17.05	12.05	16.93
Ratio of Ses:Tri	3:1	1:2	1:8
Long chain hydrocarbons (C_15_–C_23_)	10.6	-	-
Miscellaneous	0.71	2.31	4.72
Total identify	91.59	21.26	23.69

**Table 3:** Dipterocarpol content in oleo-resin and resin from different preparation processes.

Samples	Concentration (mg/g Dry Residue)	% Found
Oleo-resin	53.9 ± 2.5 ^c^	5.4 ± 0.3
Byproduct from degumming process (DG)	260.4 ± 2.9 ^a^	26.0 ± 0.3
Byproduct from distillation process (DT)	162.7 ± 1.9 ^b^	16.3 ± 0.2

Different lower-case letters indicate a significant difference of data among different samples in the same column at *p* < 0.05.

**Table 4:** Cytotoxic activity and selectivity index (SI) values of oleo-resin, resin from different preparation processes and dipterocarpol against cancer cells HepG2, HeLa, Jurkat, and in noncancerous Vero cells after 2 h exposure.

Compounds	IC_50_ (µg/mL) (Selective Index; SI)			
	Vero	HepG2	HeLa	Jurkat
Oleo-resin	88.7 ± 4.2 ^dB^	80.5 ± 1.3 (1.1) ^cB^	44. 5± 1.6 (2.0) ^bB^	23.0 ± 2.1 (3.9) ^aA^
Byproduct from degumming process (DG)	50.3 ± 0.97 ^bA^	122.9 ± 1.3 (0.4) ^cC^	43.9 ± 0.6 (1.1) ^bB^	13.0 ± 8.2 (3.9) ^aA^
Byproduct from distillation process (DT)	105.7 ± 3.4 ^dC^	88.6 ± 1.2 (1.2) ^cB^	38.3 ± 5.1 (2.8) ^bB^	15.5 ± 0.4 (6.8) ^aA^
Dipterocarpol	84.7 ± 2.5 ^cB^	24.2 ± 0.9 (3.5) ^aA^	41.1 ± 4.0 (2.1) ^bB^	>221.4 (0.4) ^dC^
Melphalan	215.6 ± 3.7 ^cD^	274.4 ± 9.1 (0.8) ^dD^	20.5 ± 0.9 (10.5) ^aA^	48.6 ± 2.2 (4.4) ^bB^

Note: Different lower-case letters indicate a significant difference of data among different cell lines in the same row at *p* < 0.05. Different upper-case letters indicate a significant difference of data among different test compounds in the same column at *p* < 0.05.

**Table 5:** Mode of cell death of oleo-resin, resin from different preparation processes and dipterocarpol at 1 × IC_50_ and 2 × IC_50_ concentrations against Jurkat cancer cells at 24 h.

Test Compounds	Test Compound Concentration (µg/mL)	Mode of Cell Death		
		Viable Cell (%)	Total Apoptosis (%)	Necrosis (%)
Control	0	80.20 ± 1.23 ^ab^	19.33 ± 1.12 ^de^	0.43 ± 0.15 ^abc^
Oleo-resin	22.96	80.67 ± 1.16 ^ab^	18.93 ± 1.16 ^e^	0.37 ± 0.06 ^abc^
	45.92	77.43 ± 0.60 ^cd^	21.87 ± 0.85 ^cd^	0.67 ± 0.29 ^a^
Byproduct from degumming process (DG)	13.04	81.03 ± 1.06 ^a^	18.77 ± 0.80 ^e^	0.23 ± 0.23 ^abc^
	26.08	79.97 ± 0.23 ^abc^	19.73 ± 0.25 ^de^	0.27 ± 0.12 ^abc^
Byproduct from distillation process (DT)	15.49	78.30 ± 0.30 ^bcd^	21.40 ± 0.36 ^cde^	0.30 ± 0.10 ^abc^
	30.98	77.30 ± 0.78 ^d^	22.50 ± 0.78 ^c^	0.20 ± 0.10 ^bc^
Dipterocarpol	13.04	79.20 ± 0.87 ^abcd^	20.50 ± 0.87 ^cde^	0.30 ± 0.00 ^abc^
	26.08	69.77 ± 0.83 ^e^	29.70 ± 1.05 ^b^	0.57 ± 0.23 ^ab^
Melphalan	48.58	77.40 ± 1.45 ^cd^	22.53 ± 1.46 ^c^	0.00 ± 0.00 ^c^
	97.16	58.37 ± 0.31 ^f^	41.30 ± 0.56 ^a^	0.00 ± 0.00 ^c^

Note: Different lower-case letters indicate a significant difference in the data among different test compounds in the same columns at *p* < 0.05.

## Data Availability

All data that support the findings of this study are available within the article.

## Supplementary Materials

The following supporting information can be downloaded at: https://www.mdpi.com/article/10.3390/molecules27103187/s1, Table S1: Chemical composition of oleo-resin and resin from different preparation processes by GC-MS analysis; Table S2: ^1^H and ^13^C-NMR data of dipterocarpol compared to the reference standard. Figure S1: The schematic diagram represents the fractionation of oleo-resin by using column chromatography to isolate dipterocarpol.

## Sample Availability

Not available.
