# Supplementary material for: Dipterocarpol in Oleoresin of Dipterocarpus alatus Attributed to Cytotoxicity and Apoptosis-Inducing Effect
Source: Molecules. 2022 May 17;27(10):3187. doi: 10.3390/molecules27103187 (PMC9145408; doi:10.3390/molecules27103187)
Supplement: Supplementary file 1 [file molecules-27-03187-s001.zip › molecules-1722317-supplementary.pdf]

Supplementary Material

Article

Dipterocarpol in Oleoresin of *Dipterocarpus alatus* Attributed to Cytotoxicity and Apoptosis-Inducing Effect

Ploenthip Puthongking [1], Chawalit Yongram [2], Somporn Katekaew [3], Bunleu Sungthong [4] and Natthida Weerapreeyakul [1,5]*

[1] Division of Pharmaceutical Chemistry, Faculty of Pharmaceutical Sciences, Khon Kaen University, Khon Kaen 40002, Thailand; pploenthip@kku.ac.th
[2] Division of Cannabis Health Science, College of Allied Health Sciences, Suansunandha Rajabhat University, Samut Songkhram 75000, Thailand; chawalit.yo@ssru.ac.th
[3] Department of Biochemistry, Faculty of Science, Khon Kaen University, Khon Kaen 40002, Thailand; somkat@kku.ac.th
[4] Integrative Pharmaceuticals and Innovation of Pharmaceutical Technology Research Unit Faculty of Pharmacy, Mahasarakham University, Maha Sarakham 44150, Thailand; bunleu.s@msu.ac.th
[5] The Research Institute for Human High Performance and Health Promotion, Khon Kaen University, Khon Kaen 40002, Thailand
* Correspondence: natthida@kku.ac.th; Tel.: +66-43-202378

Supplementary Table S1. Chemical composition of oleo-resin and resin from different preparation processes by GC-MS analysis.

Retention time	Compounds	Relative abundance (%)		
		Oleo-resin	DG	DT
**Sesquiterpenes (C_{15})**				
23.48	δ-Elemene	0.08		
24.06	α-Cubebene	0.03		
25.26	α-Copeane	0.35	0.29	
25.98	β-Elemene	0.78	0.16	
26.24	Naphthalene-	0.02		
26.88	α-Gurjunene	30.31	0.96	0.74
26.98	Caryophyllene			0.22
27.10	β-Caryophyllene	3.14		
27.47	Calarene		4.43	0.56
27.63	(-)-Isoledene	13.69		
28.27	α-Humulene	0.94		0.1
28.56	Alloaromadendrene	3.28		0.23
28.94	γ-Gurjunene	3.14		0.19
29.04	Naphthalene	0.16		
29.19	α-Amorphene	0.03		
29.28	2-Isopropenyl-4a,8-dimethyl-1,2,3,4,4a,5,6,7-octahydronaphthalene	0.28		
29.38	β-Selinene	0.16		
29.44	(-)-Dehydroaromadendrene	0.06		
29.54	(+)-Epi-bicyclosesquiphellandrene	0.56		

Retention time	Compounds	Relative abundance (%)		
		Oleo-resin	DG	DT
29.66	Viridiflorene	0.61		
29.83	α-Muurolene	0.07		
29.97	β-Vatirenene	0.12		
30.08	6-Isopropenyl-4,8a-dimethyl-3,5,6,7,8,8a-hexahydro-2(1H)-naphthalenone	0.05		
30.38	Selina-3,7(11)-diene	0.52		
30.54	δ-Cadinene	0.31		
30.83	Aromadendrene, dehydro-	0.08		
31.33	Valeranone	0.01		
31.65	Calarene epoxide	0.04		
31.86	Palustrol	0.13		
31.95	Caryophyllenyl alcohol	0.05		
32.18	Spathulenol	1.11		
32.31	(-)-Caryophyllene oxide	0.75		
32.56	(-)-Globulol	0.12		
32.86	Viridiflorol	0.14		
33.39	Rosifoliol	0.26		
33.88	α-Cadinol	0.08		
34.14	α-Copaen-11-ol	1.12		
34.69	Longifolenaldehyde	0.29		
36.36	2,2,7,7-tetramethyltricyclo[6.2.1.01,6]undec-5-en-4-one	0.05		
36.82	Isolongifolene	0.28		
36.86	Valerenal		1.06	
52.73	Longipinane	0.01		
59.17	8,9-Epoxycedrane	0.02		
**Long chain hydrocarbons (C_{15}-C_{23})**				
35.79	Myristic acid methyl ester	0.09		
37.08	9-Methyl-10,12-hexadecadien-1-ol acetate	0.06		
39.06	α-Hexylcinnamaldehyde	0.02		
39.69	Methyl palmitoleinate	0.06		
40.13	Methyl hexadecanoate	3.17		
43.39	Methyl linoleate	1.84		
43.51	Methyl oleate	4.65		
43.95	Methyl octadecanoate	0.55		
44.30	cis-9-Hexadecenal	0.09		
47.02	Methyl 11-eicosenoate	0.01		
47.44	Methyl eicosanoate	0.04		
50.64	Methyl docosanoate	0.02		
**Triterpenes (C_{30})**				
60.47	Unknown C_{30}	0.02		
61.42	Unknown C_{30}			0.26
61.45	Unknown C_{30}		1.47	
61.64	Unknown C_{30}	0.66		1.44

Retention time	Compounds	Relative abundance (%)		
		Oleo-resin	DG	DT
61.78	Otochilone	0.17		
61.86	Unknown C_{30}	0.24		
62.07	Unknown C_{30}	0.53	6.72	3.5
62.39	Unknown C_{30}	0.86		0.56
62.68	Lupenone	0.38		
63.04	Unknown C_{30}	0.65		0.56
63.50	Unknown C_{30}	0.08		
64.02	Unknown C_{30}	0.06		
64.22	Unknown C_{30}	0.07		
64.38	Unknown C_{30}	0.36		
64.92	Unknown C_{30}	0.63		
65.79	Unknown C_{30}	8.86	3.86	4.36
66.53	Unknown C_{30}	2.28		6.25
66.79	Unknown C_{30}	1.17		
67.26	Unknown C_{30}	0.03		
**Miscellaneous**				
1.04	S-Methylmethanesulfonothioate	0.1		
1.36	trans-beta-ionon-5,6-epoxide			0.1
1.40	alpha-Mercaptoacetic acid		2.31	2.53
1.51	acetone-oxime	0.06		
33.02	cyclohexane,2a,3e-dimethyl-1e,5a-divinyl-	0.28		
33.70	4-Bromo-1-naphthylamine	0.17		
34.88	Furan, 2-[(2-ethoxy-3,4-dimethyl-2-cyclohexen-1-ylidene)methyl]- (CAS)	0.05		
36.65	4a,7,7,10a-tetramethyl-dodecahydro-benzo[f]chromen-3-ylamine	0.01		
39.82	delta.9-capnellene-2.epsilon.-ol-8-one	0.04		
63.24	1-Ethyl-4,4-dimethyl-cyclohex-2-en-1-ol			0.48
65.43	Bisabolol oxide			1.61

Supplementary Table S2. ^{1}H and ^{13}C -NMR data of dipterocarpol compared to the reference standard

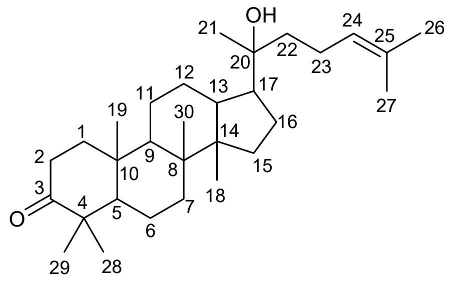

Position	In this study		Reference standard [14]	
	δ_H (ppm)	δ_C (ppm)	δ_H (ppm)	δ_C (ppm)
1-CH_2	1.83, m	39.88	1.46, ddd<br>1.92, ddd	39.88
2-CH_2	2.47, m	34.11	2.43, ddd<br>2.51, ddd	34.10
3-C	-	217.90	-	218.11
4-C	-	47.42	-	47.42
5-CH	1.29, m	55.32	1.47, m<br>1.56, m	55.33
6-CH_2	1.38, m<br>1.48, m	34.52	1.32, m<br>1.57, m	34.53
7-CH_2	1.22, m<br>1.47, m	40.26		40.26
8-C	-	40.46	1.43, dd	49.99
9-CH	1.39, m	36.81		36.81
10-C	-	19.64	1.38, dd	19.64
11-CH_2	1.43, m	22.02	1.31, m<br>1.51, m	22.02
12-CH_2	1.43, m	27.51	1.28, m<br>1.85, m	27.52
13-CH		42.36	1.66, ddd	42.36
14-C	-	50.25	-	50.25
15-CH_2	1.43, m	31.15	1.10, ddd<br>1.46, m	31.15
16-CH_2	1.20, m<br>1.78, m	24.79	1.48, m<br>1.57, m	24.79
17-CH	1.66, m	49.78	1.76, d.t.	49.79
18-CH_3	0.89, s	16.34	0.89, s	16.34
19-CH_3	0.94, s	16.03	0.94, s	16.03
20-C	-	75.35		75.34
21-CH_3	1.15, s	25.47	1.15, s	25.47
22-CH_2	1.39, m	40.46	1.47, m<br>1.49, m	40.45
23-CH_2	1.96, m	22.55	2.02, m<br>2.08, m	22.55
24-CH	5.12, m	124.68	5.12, m	124.68
25-C	-	131.63	-	131.63
26-CH_3	1.63, brs	17.72	1.63, d	17.72
27-CH_3	1.69, brs	25.76	1.69, d	25.75
28-CH_3	1.04, s	21.01	1.04, s	21.01

Position	In this study		Reference standard [14]	
	$δ_H$ (ppm)	$δ_C$ (ppm)	$δ_H$ (ppm)	$δ_C$ (ppm)
29-CH_3	1.08, s	26.69	1.08, s	26.69
30-CH_3	1.00, s	15.20	1.00, s	15.20

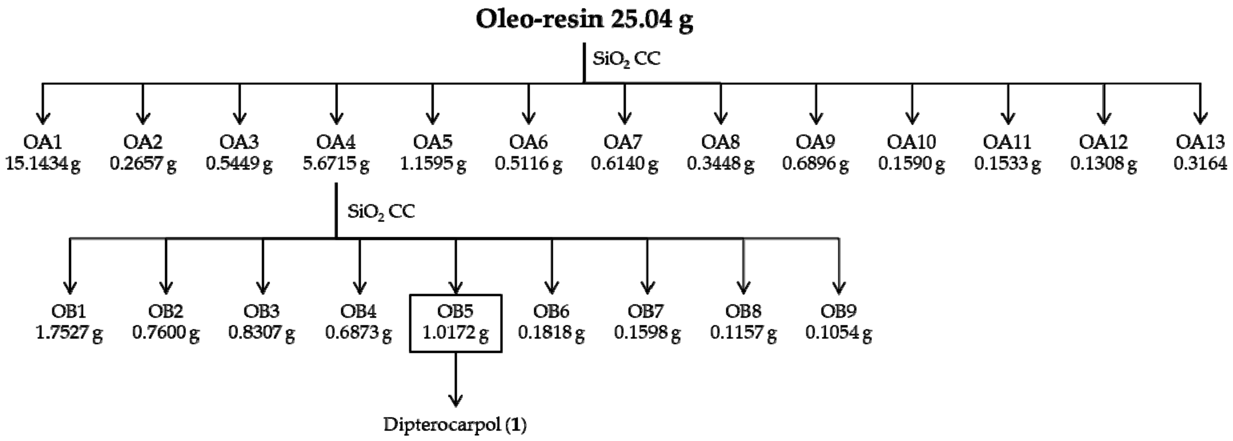

Supplementary Figure S1. The schematic diagram represents the fractionation of oleo-resin by using column chromatography to isolate dipterocarpol.
